# The Toxicity of Universal Dental Adhesives: An In Vitro Study

**DOI:** 10.3390/polym13162653

**Published:** 2021-08-10

**Authors:** Adam Wawrzynkiewicz, Wioletta Rozpedek-Kaminska, Grzegorz Galita, Monika Lukomska-Szymanska, Barbara Lapinska, Jerzy Sokolowski, Ireneusz Majsterek

**Affiliations:** 1 Department of Clinical Chemistry and Biochemistry, Medical University of Lodz, 90-419 Lodz, Poland; adam.wawrzynkiewicz@stud.umed.lodz.pl (A.W.); wioletta.rozpedek@umed.lodz.pl (W.R.-K.); grzegorz.galita@umed.lodz.pl (G.G.); 2 Department of General Dentistry, Medical University of Lodz, 92-213 Lodz, Poland; monika.lukomska-szymanska@umed.lodz.pl (M.L.-S.); barbara.lapinska@umed.lodz.pl (B.L.); jerzy.sokolowski@umed.lodz.pl (J.S.)

**Keywords:** dental materials, universal dental adhesives, biocompatibility, flow cytometry, cytotoxicity, genotoxicity

## Abstract

There is no consensus in the literature regarding the potential toxicity of universal dental adhesives (UDA). Being used in close proximity to the pulp, their biocompatibility should be an important factor in dental research. The aim of the present study was to evaluate the biocompatibility of UDA in an in vitro model. The study was performed using a monocyte/macrophage peripheral blood SC cell line (ATCC CRL-9855) on four specific UDA, namely: All-Bond Universal (Bisco); CLEARFIL Universal Bond Quick (Kuraray); G-Premio BOND (GC); Single Bond Universal (3M ESPE). The cytotoxicity of the investigated UDA was measured using the XTT colorimetric assay. The genotoxicity of the analyzed compounds was evaluated using an alkaline version of the comet assay. Furthermore, flow cytometry (FC) apoptosis detection was performed using the FITC Annexin V Apoptosis Detection Kit I. FC cell-cycle arrest assessment was performed using propidium iodide staining. The study observed significant differences in the toxicity of the UDA that were tested, as G-Premio BOND showed significant in vitro toxicity in all of the tests performed, while All-Bond Universal, CLEARFIL Universal Bond Quick and Single Bond Universal did not present any significant toxic effects toward SC cell line. The in vitro toxicity of UDA should be taken into consideration prior to in vivo and clinical studies. The flow cytometry could improve the accuracy of dental materials research and should be incorporated into the standardization criteria.

## 1. Introduction

Universal dental adhesives (UDA), despite not having a fixed definition in the literature, are commonly described as no-mix, single-bottle adhesives that can be utilized in etch-and-rinse, self-etch, and selective enamel etching bonding strategies [1,2,3,4]. UDA are versatile and can be used in both direct and indirect bonding to enamel and dentin with different materials, such as composite resins, glass ceramics, zirconia and various metals [5,6,7]. They allow the silanization step to be eliminated due to the incorporation of silane in some of their compositions [8]. UDA may have a relatively low pH, which varies from over 2.5 to 1.0, depending on the specific system [9].

UDA have similar a composition to older generation self-etch adhesives, with the main difference being the incorporation of specific carboxylate and/or phosphate functional monomers, such as 10-methacryloyloxydecyl dihydrogen phosphate (10-MDP), N-phenyl-p-phenylenediamine (Phenyl-P), 4-methacryloxyethyl trimellitic acid (4-MET) and glycero-phosphate dimethacrylate (GPDM), as well as polyalkenoic acid copolymer (PAC). These functional monomers enable the long term chemical bonding to dentin [10,11]. Additionally, UDA also contain other monomers promoting adhesion, such as dipentaerythritol pentaacrylate phosphoric acid ester (PENTA) and biphenyl dimethacrylate (BPDM), as well as hydrophobic decamethylene dimethacrylate (D3MA), hydrophilic hydroxyethul methacrylate (HEMA), amphiphilic bisphenol A-glycidyl methacrylate (bis-GMA), urethane dimethacrylate (UDMA) and triethylene glycol dimethacrylate (TEGDMA) [8]. The composition of UDA is intended to improve adhesion to various surfaces, though it may also impact the biocompatibility of the materials [12,13,14,15].

Reactions such as the inflammation of the pulp or adjacent gingiva can be observed during the close proximity of dental materials, such as resin composites and adhesives [16]. We can also observe allergic reactions or localized lichenoid reactions to dental materials, both intra- and extra-orally. In the 1970s and 1980s, the American Dental Association (ADA), as well as the Fédération Dentaire Internationale (FDI), issued a set of tests for the biocompatibility of dental materials [17]. In the following years, the safety regulations defining dental materials as medical devices were implemented in the United States of America in 1976 and in the European Union in 1993, and now exist worldwide [18].

A set of tests with various endpoints needed to be introduced to assess the biocompatibility of the materials, including the in vitro cytotoxicity and genotoxicity/mutagenicity tests, as well as sensitization tests based on in vivo animal models [19]. These properties may also be evaluated based on the amount of substances eluted from the dental materials [20]. The in vitro studies regarding genotoxicity and cellular damage are typically performed using human leukocyte cell lines [21,22,23,24]. In the most recent literature, apart from the cytotoxicity tests (such as Cell Proliferation Kit II (XTT), proposed by ISO 10993 [19]), flow cytometry (FC) is introduced as a form of evaluating the biocompatibility of dental materials [25,26,27,28]. It allows apoptosis, necrosis and cell cycle progression to be analysed [29,30].

Dental materials such as UDA should undergo a specific set of tests in order to analyze their biologic impact. In our previous study, we provided a detailed analysis of the toxicity of three other UDA: Prime&Bond Universal, Adhese Universal and OptiBond Universal [31]. The main aim of the present study is to evaluate the biocompatibility of UDA, in the highly standardized in vitro model, through the assessment of cytotoxicity and genotoxicity.

## 2. Materials and Methods

### 2.1. Universal Dental Adhesives Used in the Study

The present study investigated four UDA, namely All-Bond Universal, CLEARFIL Universal Bond Quick, G-Premio BOND and Single Bond Universal (Table 1).

### 2.2. Cell Line and Eluate Preparation

All in vitro analyses were performed with a commercially available monocyte/macrophage peripheral blood cell line—SC (ATCC CRL-9855) (ATCC; Manassas, VA, USA). Cells were maintained under standard conditions (37 °C; 5% pCO_2_; 95% humidity) according to the manufacturer’s guidelines. Cells were cultured in Iscove’s Modified Dulbecco’s Medium (IMDM) with 4-mM l-glutamine adjusted to contain 1.5 g/L sodium bicarbonate (ATCC; Manassas, VA, USA) and supplemented with 0.05-mM 2-mercaptoethanol (Sigma-Aldrich Corp., St. Louis, MO, USA), 0.1-mM hypoxanthine and 0.016-mM thymidine (90%) (ATCC; Manassas, VA, USA), fetal bovine serum (10%) (ATCC; Manassas, VA, USA) and 1% penicillin/streptomycin solution (P/S) (ScienCell Research Laboratories, San Diego ad, CA, USA). Cells were split every 2–3 days, when the cell culture reached 90–95% confluency. A total of 50 µL of each investigated UDA was placed in Eppendorf tubes and polymerized according to the manufacturer’s instructions (LED lamp intensity over 1000 mw/cm^2^, The CURE-TC-01, Spring Health Products, PA, USA). Afterwards, 1 mL of cell culture medium was added and the Eppendorfs were incubated for 24 h at 37 °C. The eluates obtained after centrifugation for 5 min at the speed of 2000 rpm were used for further experiments.

### 2.3. Cytotoxicity Analysis

To evaluate the cytotoxicity of the UDA, 2,3-Bis-(2-Methoxy-4-Nitro-5-Sulfophenyl)-2H-Tetrazolium-5-Carboxanilide (XTT) colorimetric assays were used (Thermo Scientific, Waltham, MA, USA). This test is based on the cells’ metabolism and their ability to reduce the tetrazolium salt (XTT) to an orange water-soluble formazan. Test samples were prepared in 96-well plates by adding 50 µL (8 × 10^3^ cells/well) of cell suspension and 50 µL of the tested compounds’ eluate into the complete medium. The positive control cells were suspended in 96% isopropyl alcohol, while the negative control cells were cultured in a complete medium. After 24 h incubation, 25 µL of XTT/PMS mixture was added to each well. Subsequently, after 4 h incubation, absorbance was measured at a wavelength of 450 nm using a spectrophotometer (Synergy HT, BioTek, Hong Kong, China). All of the experiments were performed in triplicate, with similar results.

### 2.4. Genotoxicity Assessment

The genotoxicity of the tested UDA was evaluated by an alkaline version of the comet assay that is used to analyze DNA damage in specific cells. Assays were prepared in 12-well plates by adding 500 µL (5 × 10^4^ cells/well) of complete medium and 500 µL of prepared eluates. Cells suspended in highly toxic 10% DMSO (Sigma-Aldrich Corp., St. Louis, MO, USA) constituted a positive control, whereas cells suspended in 1 mL of complete culture medium constituted a negative control. The incubation lasted for 24 h. Cells suspended in 0.37% LMP agarose (Sigma-Aldrich Corp., St. Louis, MO, USA) were placed on microscope slides that were previously coated with NMP agarose (Sigma-Aldrich Corp., St. Louis, MO, USA). Preparations were incubated in lysis buffer at pH 10 (2.5-M NaCl, 10-mM Tris, 100-mM EDTA), containing TritonX-100 (Sigma-Aldrich Corp., St. Louis, MO, USA), at a final concentration of 1% at 4 °C for 60 min. After 1 h incubation, the preparations were then incubated in development buffer (300-mM NaOH, 1-mM EDTA) for 20 min at 4 °C, followed by electrophoresis (32 mA, 17 V, 20 min) at 4 °C in electrophoretic buffer (30-mM NaOH, 1-mM EDTA). Subsequently, the preparations were stained with a DAPI fluorescent dye and analysed with a fluorescent microscope. The genotoxicity of the tested compounds was indicated based on the percentage of DNA in the comet tail.

### 2.5. Apoptosis Detection

Apoptotic cell death induced by the eluates of the tested compounds was assessed using an FITC Annexin V Apoptosis Detection Kit I (ApoAlert Annexin V, Clontech, CA, USA). Assays were prepared in 12-well plates by adding 500 µL (1 × 10^6^ cells/well) of complete medium and 500 µL of prepared eluates, and incubated for 24 h. Cells treated with staurosporine (Sigma-Aldrich Corp., St. Louis, MO, USA) at a concentration of 1 µM for 16 h constituted a positive control, whereas cells suspended in the complete culture medium and incubated for 24 h constituted a negative control. Subsequently, cells were washed twice with cold PBS (Sigma-Aldrich Corp., St. Louis, MO, USA) and then double stained with annexin V as a marker of early apoptosis, and propidium iodide (PI) as a marker of cell membrane disintegration, necrosis and late apoptosis. The percentage of apoptotic cells was acquired by FC using the CytoFLEX (Beckman Coulter, Brea, CA, USA). The obtained data were analyzed using the Kaluza analysis 1.5 A software (Beckman Coulter).

### 2.6. Cell Cycle Analysis

The analysis of the cell cycle was performed by FC using PI staining. Assays were prepared in 12-well plates by adding 500 µL (1 × 10^6^ cells/well) of complete medium and 500 µL of prepared eluates, and incubated for 24 h. Cells treated with 1 µM of nocodazole (Sigma-Aldrich Corp., St. Louis, MO, USA) for 16 h constituted a positive control, whereas cells cultured in a complete medium for 24 h constituted a negative control. Cells were washed twice with cold PBS (Sigma-Aldrich Corp., St. Louis, MO, USA) and then fixed with ice-cold 70% ethanol at −20 °C for 20 min. Subsequently, cells were treated with RNase A DNase&Protease-free (10 mg/mL) (Canvax Biotech, Spain) and incubated at 37 °C for 1 h before staining with PI solution (10 μg/mL) (Sigma-Aldrich Corp., St. Louis, MO, USA). After a 30-min incubation at 4 °C, the percentage of cells in each cell cycle phase was assessed using Kaluza analysis 1.5A software (Beckman Coulter). On the DNA content histograms, the number of cells was plotted on the *y*-axis, whereas the DNA content, as measured by PI fluorescence, was depicted on the *x*-axis.

### 2.7. Statistical Analysis

Statistical analysis was performed using the Sigma Plot (Systat Software, Inc., San Jose, CA, USA). The normality test was performed using a Shapiro–Wilk test. All statistical analyses, except the comet assay test, were normally distributed, therefore the statistical analysis between the two groups was performed using Student’s *t*-test. In the comet assay analysis, no normal distribution was obtained; therefore, the statistical analysis of the two groups was performed using the Mann–Whitney rank sum test. Each of the analyses in individual experiments were based on the results of three independent tests. The differences were statistically significant on the graphs as follows: * *p* < 0.05; ** *p* < 0.01; *** *p* < 0.001 versus negative controls.

## 3. Results

### 3.1. Analysis of the Cytotoxicity of the Universal Dental Adhesives

**Figure 1:**
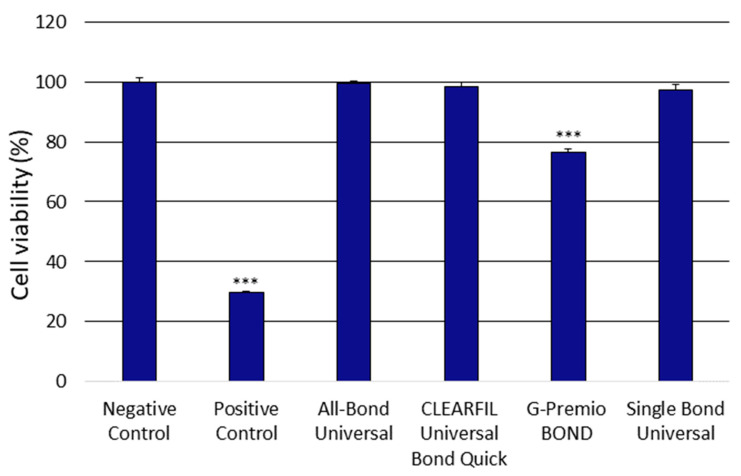
Cytotoxicity of the investigated adhesives. Test performed using the Cell Proliferation Kit II (XTT) assay after 24 h incubation of cells with the tested compounds. The positive control constituted cells were suspended in 96% isopropyl alcohol, while the negative control cells were cultured in a complete medium. The differences were statistically significant on the graphs as follows: *** *p* < 0.001 versus negative control.

The obtained XTT assay outcomes showed significant differences in the cytotoxic properties of the investigated compound eluates. The obtained results showed that only the G-PREMIO Bond significantly decreased cell viability compared to the controls used (Figure 1).

### 3.2. Analysis of the Genotoxicity of the Universal Dental Adhesives

**Figure 2:**
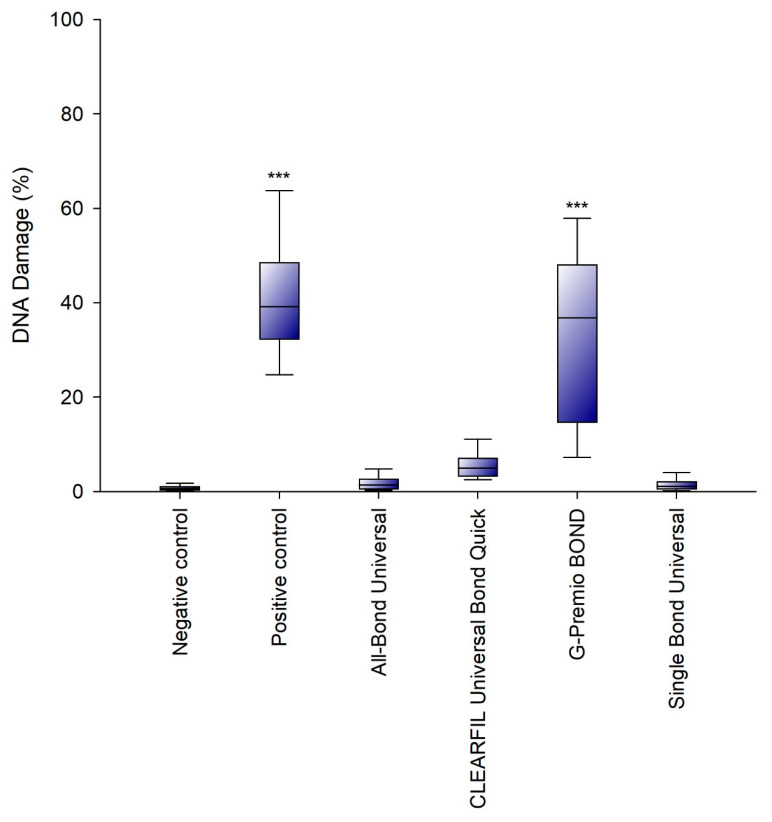
Genotoxicity of the investigated adhesives. Analysis was performed using an alkaline version of the comet assay after 24 h incubation of cells with the tested compounds. Cells suspended in 10% DMSO constituted a positive control. Cells suspended in 1 mL of complete culture medium constituted a negative control. The differences were statistically significant on the graphs as follows: *** *p* < 0.001 versus negative control.

A significant increase in DNA damage was observed after the 24 h incubation in the cells treated with G-PREMIO Bond. The other systems used did not induce significant DNA damage in the investigated SC cell line (Figure 2).

### 3.3. Apoptosis Detection by FITC Annexin V/PI Double Staining of the Universal Dental Adhesives

**Figure 3:**
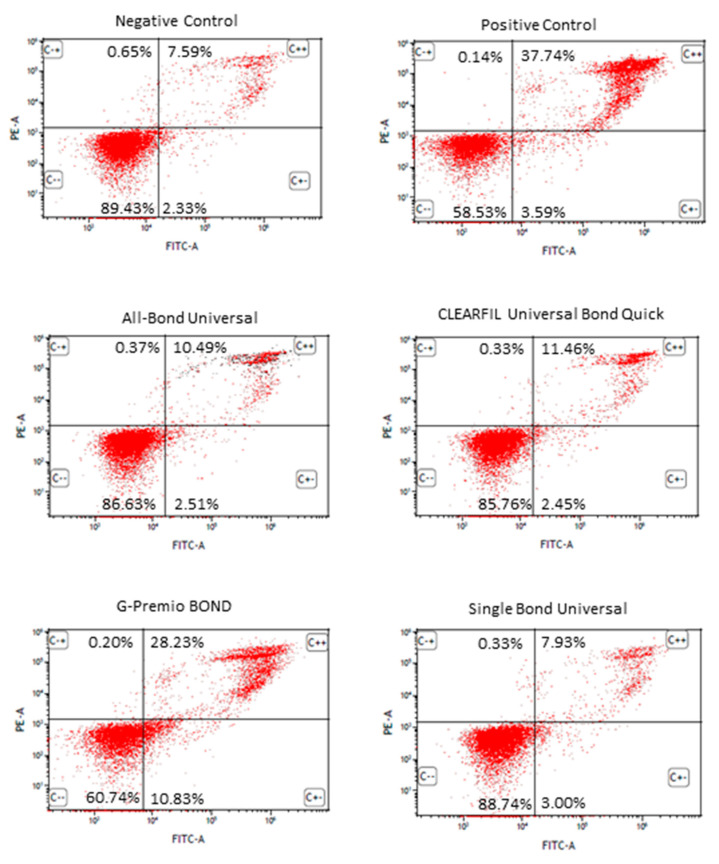
Flow cytometric FITC annexin V/propidium iodide (PI) double staining analysis of apoptosis after 24 h incubation of cells with the tested compounds. Dot plot graphs indicate the percentage of viable (FITC annexin V negative, PI negative), early apoptotic (FITC annexin V positive, PI negative) late apoptotic (FITC annexin V positive, PI positive) and necrotic (FITC annexin V negative, PI positive) cells. Cells treated with staurosporine at a concentration of 1 µM constituted a positive control. Cells suspended in the complete culture medium constituted a negative control.

After treatment with staurosporine, a significant number of SC cells underwent apoptosis in comparison to the control cells only incubated with the complete cell culture medium. After a 24 h incubation, G-Premio Bond significantly induced apoptosis (approximately 39% of cells were at the early and late stages of apoptosis). The other UDA did not significantly induce apoptosis in the SC cell line tested. Additionally, none of the tested compounds evoked a significant increase in the level of necrotic SC cells (Figure 3).

### 3.4. Analysis of the Cell Cycle Progression by PI Staining of the Universal Dental Adhesives

**Figure 4:**
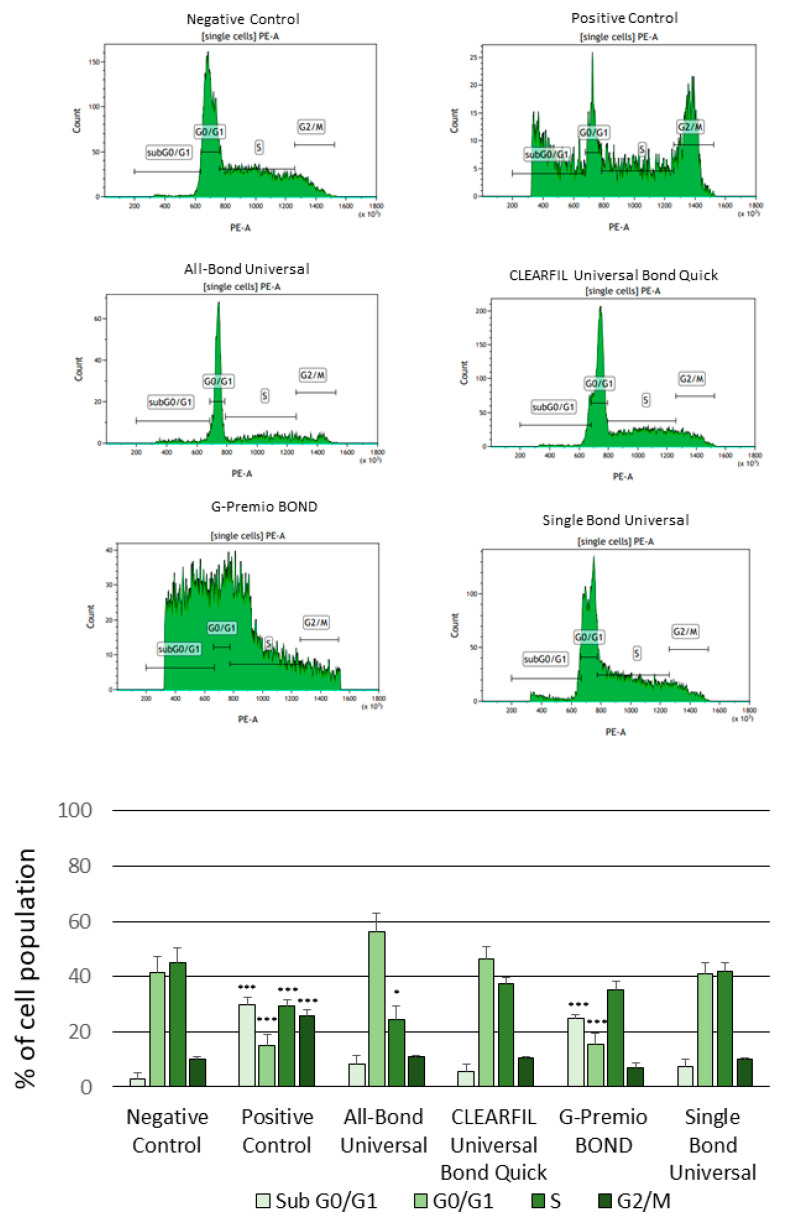
Flow cytometry (FC) analysis of cell cycle progression using the propidium iodide (PI) staining after 24 h incubation of cells with the tested compounds. Cells treated with 1 µM nocodazole constituted a positive control. Cells cultured in the complete medium constituted a negative control. The differences were statistically significant on the graphs as follows: * *p* < 0.01, *** *p* < 0.001 versus negative control.

The cell cycle progression of the SC cells treated with the investigated compounds was similar to the SC cells cultured in the complete medium, except that the obtained results demonstrated an arrest in the G2/M phase of the SC cell cycle treated with nocodazole used as a positive control. The G-Premio Bond triggered a significant increase in the percentage of SC cells in the sub-G0/G1 phase and a significant decrease in the percentage of cells in the G1 phase of the cell cycle as compared to the negative control. Moreover, we noticed a decrease in the percentage of SC cells treated with All-Bond Universal in the S phase of the cell cycle (Figure 4).

## 4. Discussion

In the present study, all of the UDA tested showed significantly different toxicity. While All-Bond Universal, CLEARFIL Universal Bond Quick and Single Bond Universal showed no significant cytotoxicity and genotoxicity, according to the controls used, G-Premio BOND significantly decreased the cell viability and presented significant DNA damage in the comet assay. Additionally, similar results occurred in the apoptosis detection test performed using FC. All-Bond Universal, CLEARFIL Universal Bond Quick and Single Bond Universal did not show significant increases in apoptosis in the tested cell line, while G-Premio BOND significantly increased the percentage of cells in the early and late stages of apoptosis. The cell cycle analysis with FC showed a significant increase in the sub-G0/G1 phase in cells that were treated with the G-Premio BOND eluate, which indicates that there was a higher number of dead cells compared to the controls used and the other UDA tested.

The results of the present study showed consistency in all the tests conducted and were in line with the results we previously obtained for other UDA, namely: Prime & Bond Universal (Dentsply Sirona, Charlotte, NC, USA); OptiBond Universal (Kerr, Brea, CA, USA); Adhese Universal (Ivoclar Vivadent, Schaan, Liechtenstein) [31]. These differences in toxicity could be the result of differences in the composition of the specific UDA, namely, different monomers and the ratio of the compounds.

It was stated that dental adhesives may incorporate different concentrations of methacrylate monomers, such as TEGDMA, UDMA, HEMA, PENTA and bis-GMA, into their composition, which may impact their toxicity. The synergistic interactions between them may also result in the amplification of their toxic effect in comparison to the individual monomers [33].

Conservative dental intervention is used to preserve the pulp and avoid the possible adverse effects caused by restorative biomaterials in dentistry. Biocompatibility is one of the most important properties of dental materials. It is especially crucial in materials that have direct or indirect contact with the pulp or oral soft tissues. Dental adhesives should be screened for biocompatibility using highly standardized techniques and tests that include the different mechanisms of potential cytotoxicity, genotoxicity, apoptosis induction or cell cycle arrest, as there is no consensus on their effects on human tissues.

Other studies have revealed that bis-GMA showed the highest cytotoxicity among the methacrylate monomers in comparison to UDMA, HEMA and TEGDMA, which showed less cytotoxicity according to the literature [33,34,35,36,37]. Among the monomers used in dental adhesives, bis-GMA has relatively high cytotoxicity. This monomer has a low ability to penetrate dentin because of its high molecular weight, but is susceptible to hydrolysis, and its products may induce the loss of cell membrane permeability [38,39]. It was reported that bis-GMA may provoke prostanoid production, leading to cytotoxicity in pulp cells, which could result in inflammation or pulpal necrosis due to the production of reactive oxygen species (ROS) [40]. Additionally, it was stated that in human gingival fibroblasts treated with bis-GMA, there was a depletion of intracellular glutathione (GSH) and the induction of apoptosis [41]. UDMA toxicity is induced via GSH depletion, cell cycle arrest, apoptosis/necrosis induction and ROS production [42]. It also amplifies the mRNA expression of carboxylesterase-2, heme oxygenase-1, cyclo-oxygenase-2 and in the cells present in pulp [43].

HEMA is capable of rapid diffusion through dentin [44] and can result in toxicity in the cells present in pulp tissue. It has been stated that it can induce morphological changes in several types of cells, and can induce apoptosis and growth suppression [45,46].

Functional acidic monomers are an important part of UDA composition due to their adhesive properties. As the most commonly used, 10-MDP showed suppressed odontoblastic differentiation of human pulp cells, as well as an inflammatory response [47]. It is also capable of mineralization depression through direct interaction with odontoblast-like cells [48].

In comparison, according to Nakagawa et al., testing 4-MET in luting materials containing resin showed biocompatibility in the cells present in dental pulp [49,50]. Additionally, it was reported that the lower pH values may result in higher toxicity of the dental adhesives [51].

Apart from the monomers, the photo-initiators, such as camphorquinone or diphenyl (2,4,6-trimethylbenzoyl) phosphine oxide, when added to the dental adhesives, may also elute from the dental resin materials in vitro and result in cytotoxicity towards cells [52].

The limitations of the present study revolve around the in vitro character of the experiments. The credibility of the methods used is high, as we acquired similar results in different tests based on different molecular mechanisms of toxicity, cytotoxicity, genotoxicity, apoptosis induction and cell cycle arrest, and the results were comparable to our previous study based on different UDA using a similar methodology [31]. Light-curing parameters, such as light intensity and type, distance and curing mode, may all influence the polymerization of dental adhesives, and therefore the amount of residual monomers and their toxicity [53,54]. Additionally, the absence of a dentin barrier, smear layer and immune response, as well as the fact that UDA are present in the tooth structure for several years, also means that the in vitro results cannot be freely compared to a clinical situation present in a tooth cavity [55,56].

## 5. Conclusions

The results presented in this manuscript showed that the UDA that were investigated have different impacts on the SC cell line. Indicated toxicity variation should be taken into consideration in further clinical studies.

Moreover, we suggest that the methods used in dental materials research should be extended to include different tests, such as comet assay and flow cytometry apoptosis detection, as well as cell-cycle arrest evaluation. In particular, tests based on flow cytometry could improve the accuracy of the research and should be incorporated into the standardization criteria. The in vitro toxicity of the UDA should be taken into consideration and monitored as a key safety marker for the assessment of dental materials prior to clinical studies.

## Figures and Tables

**Table 1:** Universal dental adhesives used in the study.

Name	Manufacturer	Lot Number	Composition
All-Bond Universal	Bisco, Inc. Schaumburg, IL 60193, USA	2000000048	bis-GMA (20–50%), Ethanol (30–50%), 10-MDP (5–25%), HEMA (5–25%) [14]
CLEARFIL Universal Bond Quick	Kuraray Europe GmbH, 65795 Hattersheim am Main, Germany	CL0201	bis-GMA (10–25%), ethanol (10–25%), HEMA (2.5–10%), Other ingredients: 10-MDP, Hydrophilic amide monomers, Colloidal silica, Silane coupling agent, Sodium fluoride, dl-Camphorquinone, Water [27]
G-Premio BOND	GC EUROPE, 3001 Leuven, Belgium	1910251	ethyl alcohol (35–50%), 2,2’-[(4-methylphenyl)imino]bisethanol (5–10%) Other ingredients: 4-MET, 10-MDP, MDTP [8]
Single Bond Universal	3M ESPE Dental Products, 3M Center, St. Paul, MN 55144-1000, USA	00305A	bis-GMA (15–25%), HEMA (15–25%), D3MA (5–15%), silane treated silica (5–15%), ethanol (10–15%), water (10–15%), 2-propenoic acid, 2-methyl-, reaction products with 1,10-decanediol and phosphorous oxide (P_2_O_5_) (1–10%), copolymer of acrylic and itaconic acid (1–5%), dimethylaminobenzoat(−4) (<2%), (dimethylamino)ethyl methacrylate (<2%), camphorquinone (<2%), methyl ethyl ketone (<2%) [32]

## Data Availability

The data presented in this study are available on request from the corresponding author.
